# Polysulfone/MMT Clay Mixed Matrix Membranes for Efficient Diclofenac Removal and Improved Antifouling Performance in Wastewater Treatment

**DOI:** 10.3390/membranes15110344

**Published:** 2025-11-18

**Authors:** Zouhair Salah, Hajer Aloulou, Catia Algieri, Lasaad Dammak, Raja Ben Amar

**Affiliations:** 1 Research Unit ‘Advanced Technologies for Environment and Smart Cities’, Faculty of Sciences of Sfax, University of Sfax, Sfax 3000, Tunisia; zouhair.salah.etud@fss.usf.tn (Z.S.); hajer.aloulou89@yahoo.fr (H.A.); 2 Department of Chemical, Preparatory Institute for Engineering Studies of Gabes, University of Gabes, Gabes 6029, Tunisia; 3 Institute on Membrane Technology, National Research Council of Italy (ITM–CNR), Cubo 17C, Via Pietro Bucci, 87036 Rende, Italy; 4 Institut de Chimie et des Matériaux Paris Est, ICMPE UMR-CNRS 7182-UPEC, Université Paris-Est Créteil, 2 rue Henri Dunant, 94320 Thiais, France

**Keywords:** montmorillonite, polysulfone, mixed-matrix membrane, diclofenac removal, antifouling

## Abstract

Due to industrialization and globalization, water sources are increasingly contaminated with drugs. Among the various methods available, adsorption remains one of the most widely used techniques for drug removal. This work was to develop polysulfone (PSF) membranes integrated with montmorillonite (MMT) clay. The fabricated membranes were subsequently evaluated for their performance in removing diclofenac (DCF) from aqueous solutions. The membranes were characterized using scanning electron microscopy (SEM), energy-dispersive X-ray spectroscopy (EDX), Fourier transform infrared spectroscopy (FTIR), contact angle measurements, as well as chemical and mechanical tests. Adding MMT at 1.5 and 2 wt% improved both hydrophilicity and mechanical strength. The natural hydrophilicity of MMT also accelerates the non-solvent/solvent exchange during phase inversion, resulting in higher porosity. These structural and surface modifications increased water permeability (16.36 L·m^−2^·h^−1^·bar^−1^), achieved 79% DCF removal, and enhanced antifouling properties. However, increasing the MMT clay content to 2.5 wt% caused particle aggregation, which reduced membrane performance. Fouling resistance tests with bovine serum albumin (BSA) as a model foulant showed a rejection rate of 89% and a flux recovery ratio (FRR) above 82% using an optimized membrane. These findings demonstrate that PSF/MMT membranes can serve as promising candidates for sustainable pharmaceutical wastewater treatment.

## 1. Introduction

Today, access to fresh and clean water remains one of the most pressing global challenges, drawing considerable attention from the scientific community [1]. Numerous pollutants found in aquatic environments exert harmful effects on ecosystems and human health, posing serious threats to the survival of marine organisms and other life forms [2]. Among these, pharmaceutical residues are particularly concerning, as they represent a major class of emerging contaminants discharged into water bodies, primarily from pharmaceutical industries [3,4]. Additional sources include biomedical waste from hospitals, human excreta, livestock treatments, and veterinary medicines [5,6]. DCF is widely recognized as a priority emerging contaminant by several international environmental agencies because of its persistence, limited biodegradability, and ecotoxicological effects on aquatic organisms and even terrestrial wildlife. It is one of the most commonly detected non-steroidal anti-inflammatory drugs (NSAIDs) in water bodies. It has become a model compound in numerous membrane-based and adsorption studies for pharmaceutical removal [7]. It is frequently detected in aqueous effluents [8]. Concentrations of DCF have been reported in municipal wastewater at levels reaching up to 57.16 µg·L^−1^ [9,10]. This compound is associated with several adverse effects, including chronic headaches, gastric pain, chest discomfort, respiratory difficulties, fever, and allergic reactions [10]. Consequently, its removal from water has become a priority for researchers. Several treatment methods have been investigated for the elimination or degradation of pharmaceutical pollutants, including adsorption [11], ozonation [12], bio-sorption, advanced oxidation processes [13,14], coagulation and flocculation [15], and membrane processes [16,17].

Among various treatment techniques, the use of adsorbents for removing low concentrations of hazardous contaminants has shown superior performance compared to alternative methods [18]. Activated carbon is the most commonly employed commercial adsorbent; however, its high cost restricts large-scale applications [19]. To overcome these limitations, organic–inorganic hybrid materials have recently been developed to fabricate membranes with enhanced properties and broader application potential [15,20]. For instance, Salah et al. incorporated graphene oxide (GO) into a polysulfone (PSF) matrix to improve both water permeability and diclofenac (DCF) removal [21], while Chai et al. embedded Fe_3_O_4_/GO into PSF to enhance permeability and humic acid rejection [22]. A wide range of inorganic particles, including metal oxides, zeolites, and clays, has been introduced into membrane fabrication [23,24]. Among these, clay has attracted growing interest as a filler due to its low cost, abundance, environmental friendliness, and ability to improve polymer properties even at low loading levels significantly. Polymer–clay composites have been applied in numerous industrial, technological, and environmental fields [25], particularly in water treatment, where clay improves hydrophilicity and enhances pollutant removal efficiency [25].

Clay is considered one of the most promising fillers for membrane modification, owing to its high specific surface area and intrinsic hydrophilicity, and is the principal factor in improving membrane performance [26]. Montmorillonite (MMT), a layered aluminosilicate clay with a basal spacing of approximately 1 nm, is of particular interest [27]. Structurally, MMT consists of alternating layers of silica in tetrahedral coordination and alumina in octahedral coordination [28].

Despite its potential, relatively few studies have reported on PSF–clay nanocomposites. Notably, two works employed the solution dispersion method with organophilic MMT as a filler [29,30], while another study investigated a PSF/cyanate ester/organophilic MMT blend [31]. Beyond PSF, polymer–clay nanocomposite (PCN) membranes have been widely explored in the context of fuel cells, particularly using Nafion combined with either protonated MMT [32] or sulfonated MMT [33]. Other combinations include sulfonated poly (ether ketone) with both organophilic and sulfonated MMT [34] as well as organophilic MMT blended into polyamide, poly (vinylidene fluoride), and poly-lactic acid, yielding membranes with improved physicochemical properties [35].

In this study, polysulfone–montmorillonite (PSF–MMT) mixed matrix membranes (MMMs) were prepared using the non-solvent induced phase separation (NIPS) method. The effect of MMT clay incorporation on the membranes’ hydrophilicity, mechanical strength, water permeability, and morphology was systematically evaluated. Membrane performance was tested through the removal of diclofenac from water solution, while bovine serum albumin (BSA) served as a model foulant to assess the antifouling properties and flux recovery of the fabricated nanocomposite membranes.

**Scheme 1:**
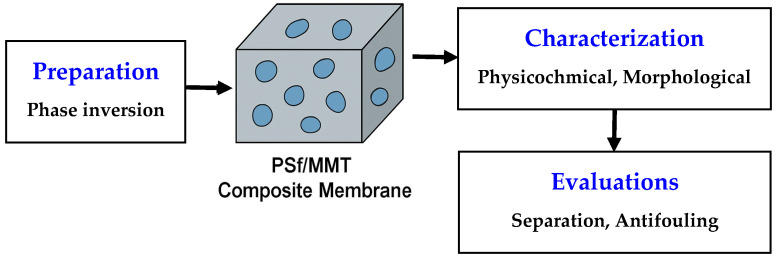
Conceptual representation of the PSF/MMT membrane development process, from fabrication to performance assessment for diclofenac removal.

Scheme 1 presents the conceptual framework of this work. It highlights the sequential approach adopted in this study: (i) fabrication of the PSF/MMT composite membranes through the phase inversion method, (ii) characterization of their physicochemical and morphological properties, and (iii) evaluation of their separation and antifouling performance. This workflow provides a clear overview of the experimental logic linking membrane structure to performance.

## 2. Methodology

### 2.1. Chemicals and Reagents

Polysulfone (PSF, average molecular weight Mw = 22,000 g·mol^−1^) and N-methyl-2-pyrrolidone (NMP, 99.7%) were purchased from Merck (Darmstadt, Germany). NMP was used directly as a solvent for preparing the polymer solution. Montmorillonite clay (MMT) was purchased from Sigma-Aldrich (St Louis, MO, USA). Distilled water was used both as a non-solvent during membrane fabrication and for water flux measurements. Sodium hydroxide (NaOH), ethanol (C_2_H_5_OH), and diclofenac sodium (DCF, C_14_H_10_Cl_2_NNaO_2_) were also purchased from Sigma-Aldrich.

### 2.2. Membrane Preparation

**Figure 1:**
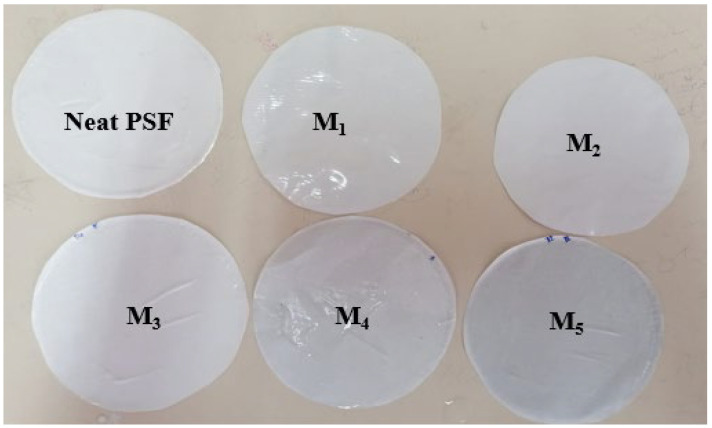
Image of the different prepared membranes.

Two types of membranes were fabricated: one based on pure polysulfone (PSF) and the other on polysulfone incorporated with montmorillonite (PSF/MMT) clay. Using the non-solvent induced phase separation (NIPS) method [36], a homogeneous polymer solution was first prepared by dissolving PSF in NMP at the specified weight ratio, under continuous stirring at room temperature until the PSF pellets were completely dissolved. Then, the required amount of MMT powder was added and dispersed under stirring at 400 rpm for at least 6 h, until a visually uniform suspension was attained. The casting solution was evenly spread onto a clean glass plate using a manual casting knife set at a thickness of 250 μm. The cast film was exposed to air for 20 s before being immersed in a coagulation bath containing distilled water at 25 °C. The glass plate was kept in the bath for 10 min to ensure complete phase inversion. The membranes obtained were thoroughly rinsed with deionized water multiple times to remove residual solvent and then dried at room temperature. Finally, they were stored in fresh distilled water before use. The compositions of PSF, NMP, and MMT are summarized in Table 1. Representative photos of the prepared membranes are shown in Figure 1.

### 2.3. Characterization Techniques

Structural characterization of MMT powder and the prepared membranes was carried out with an X-ray diffractometer (Rigaku MiniFlex 600, Tokyo, Japan) equipped with CuKα radiation (λ = 1.5406 Å) operated at 40 kV and 20 mA. Powdered samples were scanned in the 2θ range of 5–50°, with a step size of 0.02° and a scanning rate of 1°·min^−1^.The MMT powder morphology was evaluated by scanning electron microscope (SEM) using a Zeiss LEO 400 microscope (Carl Zeiss Microscopy Ltd., Cambridge, UK).

Functional groups present on the powder and the different membranes were investigated using Fourier transform infrared spectroscopy (Spectrum Two FT-IR Spectrometer, Perkin-Elmer, Waltham, MA, USA).

The surface (top-view) and cross-sectional morphologies of the membranes were examined using a field-emission scanning electron microscope (FE-SEM, ZEISS Crossbeam 350, Jena, Germany, S/N-8405010133). Cross-sections were prepared by immersing the membranes in liquid nitrogen and then fracturing them to preserve the original structure. To prevent charging during SEM observation, the samples were sputter-coated with a thin layer of platinum. Energy-dispersive X-ray spectroscopy (EDS) with an accelerating voltage of 15 kV, performed on a ZEISS Crossbeam 350 UHR-SEM instrument.

The surface hydrophilicity of the membranes was assessed using static water contact angle measurements. All samples were dried in an oven at 50 °C for 4 h before testing. Measurements were performed with a CAM 200 Optical Contact Angle Meter (KSV Instruments, Helsinki, Finland) at five different points on each membrane surface. For each sample, four measurements were performed, and both the average value and the standard deviation were calculated.

The mechanical strength of the membranes was characterized using a universal testing machine (Zwick/Roell, BTC-FR2. 5TN.D09, Ulm, Germany). Tests were performed at 25 °C with a tensile speed of 10 mm·min^−1^ and a clamping distance of 25 mm. For each membrane type, a minimum of five measurements were conducted to ensure reproducibility and reduce experimental variability.

To examine the effect of MMT on membrane structure, porosity was measured using the gravimetric method. First, the membranes were dried at 50 °C for 24 h. They were then cut into small pieces (1 cm × 1 cm), with five pieces prepared for each membrane, and their dry weights were recorded. The samples were then immersed in distilled water at 25 °C for 24 h. After removing surface water with a paper filter, the wet weight of each piece was measured. The average dry and wet weights for each membrane were used to calculate porosity (*ε*) according to Equation (1) [37].

(1) $$\varepsilon \;(\%)=\frac{\frac{W_{1}-W_{2}}{\rho_{w}}}{\frac{W_{1}-W_{2}}{\rho_{w}}+\frac{W_{2}}{\rho_{m}}}\times 100$$

where *W*_1_ and *W*_2_ are, respectively, the wet and dry weights of the membrane (g). *ρ_w_* is the density of distilled water (0.998 g·mL^−1^ at 25 °C), and *ρ_m_* is the density of the polymer (1.24 g·mL^−1^ at 25 °C).

The average pore radius (*r_m_*) of the membranes was estimated using the Guerout–Elford–Ferry Equation (2) [38].

(2) $$r_{m}=\sqrt{\frac{\left(2.9-1.75\;\;\varepsilon \right)\times 8\;\eta \;l\;Q}{\varepsilon \;A\;\Delta P}}$$

where *r_m_* is the mean pore radius (m), *η* is the water viscosity (8.9 × 10^−4^ Pa·s), *l* is the membrane thickness (m) measured using a micrometer, *Q* is the volume of permeate water per unit time (m^3^·s^−1^), *A* is the effective area of the membrane (m^2^), and Δ*P* is the operating pressure (Pa).

The chemical stability of the synthesized membranes was assessed by tracking their weight loss over time. Membrane samples were soaked in acidic (nitric acid, HNO_3_, 0.2 M, pH = 2) and alkaline (sodium hydroxide, NaOH, 0.5 M, pH = 12) solutions at room temperature for three days. The weight of each membrane was measured at regular intervals to detect any degradation or chemical damage caused by the media.

### 2.4. Filtration Tests

**Figure 2:**
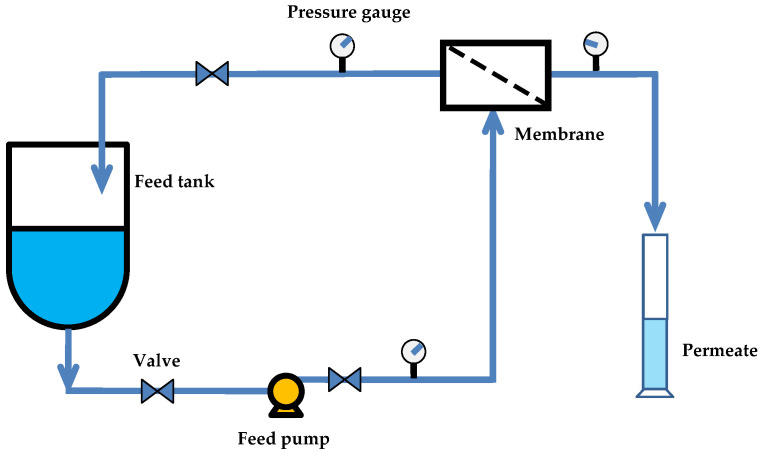
Schematic diagram of the crossflow filtration unit used.

The permeation experiments were conducted using a crossflow filtration system (Figure 2). Before testing, each membrane was compacted by circulating distilled water under a transmembrane pressure of 10–20 bar for 2 h to eliminate structural relaxation effects and to ensure steady-state flux conditions. All filtration experiments were subsequently performed in crossflow mode at 25 °C under various transmembrane pressures (Δ*P* = 10–20 bar). The pure water flux for each membrane was calculated using Equation (3) [31]:

(3) $$J=\frac{V}{A\times t}$$

where *J* is the permeating flux through the membrane, *V* is the permeate volume collected during the time *t*, and *A* is the membrane area (effective membrane area: 15.2 cm^2^). The permeability was determined by plotting the slope of the flux versus the Δ*P*. Subsequently, an aqueous solution of DCF was prepared in distilled water at a concentration of 20 mg·L^−1^ under 20 bar and 25 °C.

The permeate was collected every 10 min, and the quantitative determination of the drug was carried out using a UV spectrophotometer (UV–3100 PC Spectrophotometer, Hangzhou Boyn Instrument Co., Ltd., Hangzhou, China) at a wavelength of 273 nm. The rejection was calculated as follows [37]:

(4) $$R(\%)=\frac{C_{i}-C_{f}}{C_{i}}\times 100$$

where *C_i_* and *C_f_* are the concentrations of the drug (mg·L^−1^) in permeate and feed, respectively.

### 2.5. Fouling Studies Test

The flux recovery ratio (FRR) quantifies the ability of the membrane to recover its permeability after filtration and cleaning. In this case, the antifouling performance of the membrane was evaluated using a bovine serum albumin (BSA) solution (50 mg·L^−1^). The process involved three main steps:

- Pure water filtration at 20 bar to obtain the initial pure water flux (*J_w_*);
- BSA solution filtration;
- Cleaning the membrane with distilled water to remove reversible foulants, followed by measuring the recovered pure water flux (*J_wa_*).

The *FRR* of the membrane was then estimated using Equation (5) [39]:

(5) $$FRR(\%)=\frac{J_{wa}}{J_{w}}\times 100$$

## 3. Results and Discussion

### 3.1. Characterization of Montmorillonite (MMT)

**Figure 3:**
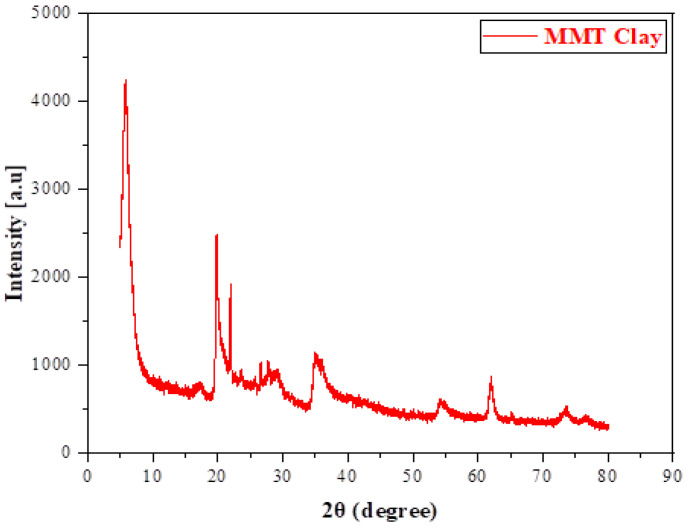
XRD patterns of montmorillonite (MMT) clay.

The XRD pattern of MMT clay (Figure 3) shows a strong reflection at 2θ = 9.9°, corresponding to the (001) basal plane with an interlayer spacing of approximately 8.8 Å. This spacing is slightly smaller than the typical hydrated montmorillonite value (12–15 Å), indicating a partially dehydrated or collapsed structure [40]. Additional reflections at higher angles, such as 20°, 22°, 35°, and 62°, can be attributed to higher-order reflections as well as the presence of crystalline impurities, mainly quartz and feldspar, that are commonly associated with natural clays. Moreover, the broad background hump between 20° and 35° suggests the presence of partially amorphous phases or structural disorder within the clay [41]. Overall, the diffraction profile confirms the crystalline fingerprint of montmorillonite, reflecting both its ordered layered structure and the contribution of minor impurities and amorphous components.

**Figure 4:**
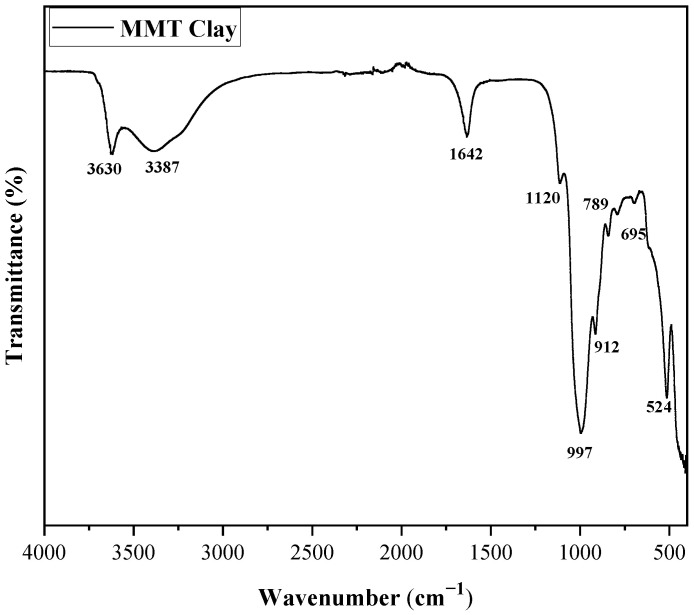
FTIR spectrum of montmorillonite (MMT) clay.

The FTIR spectrum of MMT clay, shown in Figure 4, displays characteristic vibrational bands linked to its layered silicate structure. The broad absorption peaks of 3630 cm^−1^ and 3387 cm^−1^ are assigned to O–H stretching vibrations from structural hydroxyl groups and adsorbed water molecules, respectively [42]. A distinct band at 1642 cm^−1^ is attributed to the bending vibrations of water molecules in the interlayer spaces [43]. In the lower wavenumber region (1200–400 cm^−1^), the strong absorption at 1120 cm^−1^ corresponds to the Si–O stretching vibration of tetrahedral silicate sheets, while the peak at 997 cm^−1^ relates to the in-plane Si–O stretching mode, confirming the integrity of the layered silicate framework [44]. Peaks at 789 cm^−1^ and 695 cm^−1^ are linked to Al–Mg–OH and Al–OH bending vibrations, respectively [45]. The low-wavenumber band around 524 cm^−1^ is assigned to Si–O–Al bending modes. Overall, the FTIR profile verifies the typical structural features of montmorillonite clay, indicating its preserved layered structure with characteristic hydroxyl and silicate functionalities, as reported in previous studies [43,45].

**Figure 5:**
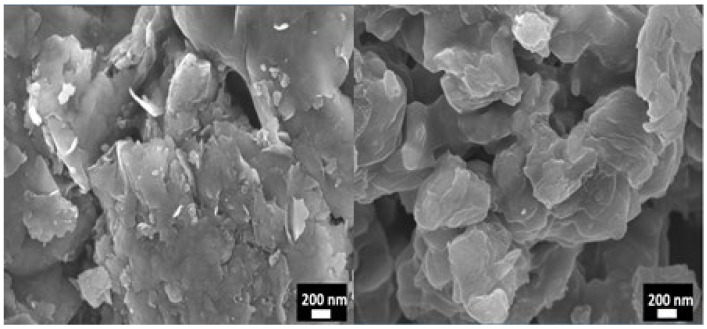
SEM images of MMT clay.

Figure 5 shows well-defined platelets with nanoscale thickness forming a loosely stacked lamellar structure. The uniform dispersion of platelets and the textured surface indicate the clay’s high surface area and its potential for adsorption, intercalation, and incorporation into nanocomposites [46]. These morphological features corroborate the lamellar structure observed by XRD and the integrity of the layered silicate framework revealed by FTIR, providing complementary evidence of the preserved structural characteristics of the commercial MMT clay.

### 3.2. Membrane Characterization

#### 3.2.1. XRD Analysis

**Figure 6:**
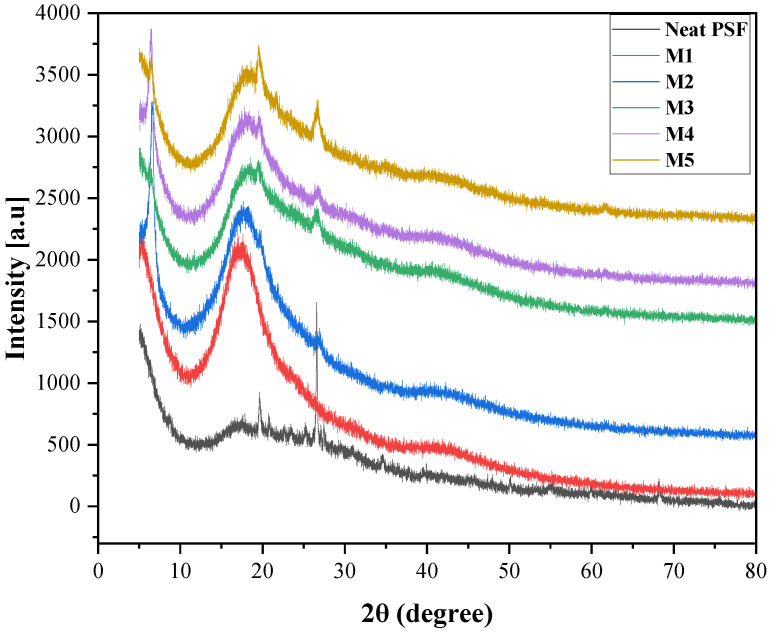
XRD pattern of pristine and mixed matrix membranes: M_1_ (PSF/MMT-0.5%), M_2_ (PSF/MMT-1%), M_3_ (PSF/MMT-1.5%), M_4_ (PSF/MMT-2%), and M_5_ (PSF/MMT-2.5%).

To investigate the structural properties and verify the successful incorporation of montmorillonite (MMT) clay into the polysulfone (PSF) matrix, X-ray diffraction (XRD) analyses were performed on the pristine and mixed matrix membranes, as shown in Figure 6.

The diffraction pattern of neat PSF shows a broad amorphous halo, characteristic of its non-crystalline structure. When MMT is incorporated at different loadings, noticeable changes appear in the diffraction profiles. At low clay concentrations (M_1_ and M_2_), the disappearance or broadening of the characteristic basal reflection of MMT indicates exfoliation and/or intercalation of silicate layers within the PSF matrix. This suggests good dispersion of the clay, with polymer chains penetrating between clay platelets.

As the MMT content increases (M_3_–M_5_), the intensity of certain reflections becomes more pronounced, indicating the partial restacking or aggregation of clay platelets at higher loadings. However, the overall amorphous nature of PSF is preserved, confirming that no significant crystalline domains are formed.

These findings demonstrate that MMT was successfully integrated into the PSF matrix, with predominantly exfoliated and intercalated structures at low loadings, whereas higher concentrations tend to cause partial aggregation. These structural changes are likely to impact the physical and functional properties of the membranes, including their hydrophilicity, permeability, and mechanical strength [43,47].

#### 3.2.2. FT-IR Studies

**Figure 7:**
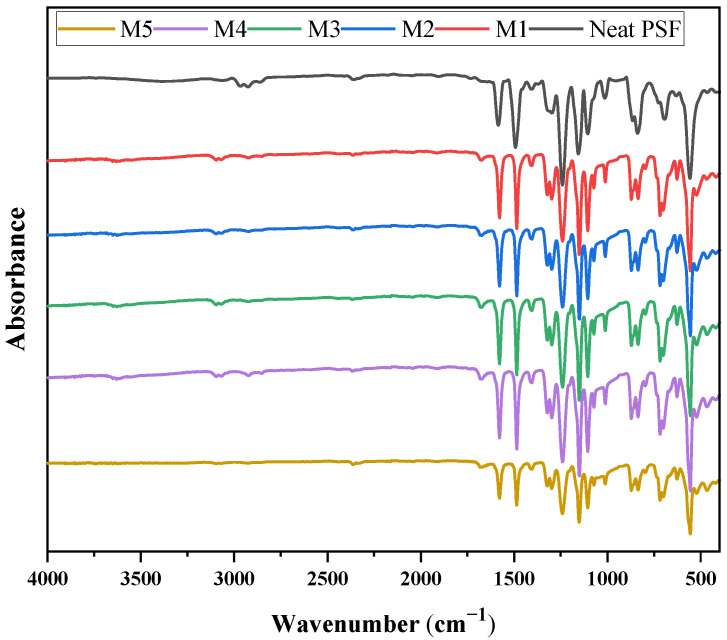
FTIR spectra of the different prepared membranes.

Figure 7 displays the FTIR spectra of the pristine polysulfone (PSF) membrane and the PSF–MMT membranes. The spectrum of pristine PSF exhibits the characteristic bands of the polymer: aromatic C–H stretching at 3100–3000 cm^−1^, aliphatic C–H stretching at 2920 cm^−1^, aromatic C=C vibrations around 1585 cm^−1^, and the strong sulfone (S=O) absorptions at 1320–1290 cm^−1^ (asymmetric) and 1150–1130 cm^−1^ (symmetric). In addition, the ether (C–O–C) stretching band is observed near 1240 cm^−1^, confirming the polysulfone backbone [48]. After the incorporation of montmorillonite, new peaks appeared: a broad absorption in the 3200–3600 cm^−1^ region, associated with surface hydroxyl groups and interlayer water of the clay (M_3_, M_4,_ and M_5_), and a strong band in the 1000–1100 cm^−1^ region, attributed to Si–O stretching vibrations of the silicate layers [49]. Slight broadening and shifts in the characteristic PSF bands, especially those corresponding to S=O and C–O–C, indicate interfacial interactions (hydrogen bonding and ion–dipole contacts) between the oxygen-containing groups of PSF and the hydroxyl sites of MMT [50]. As shown in Figure 6, the absence of new functional bands suggests that MMT incorporation occurs primarily through physical interactions rather than covalent bonding, which is consistent with a previous study on PSF–MMT hybrid membranes [51].

#### 3.2.3. Morphological Studies

**Figure 8:**
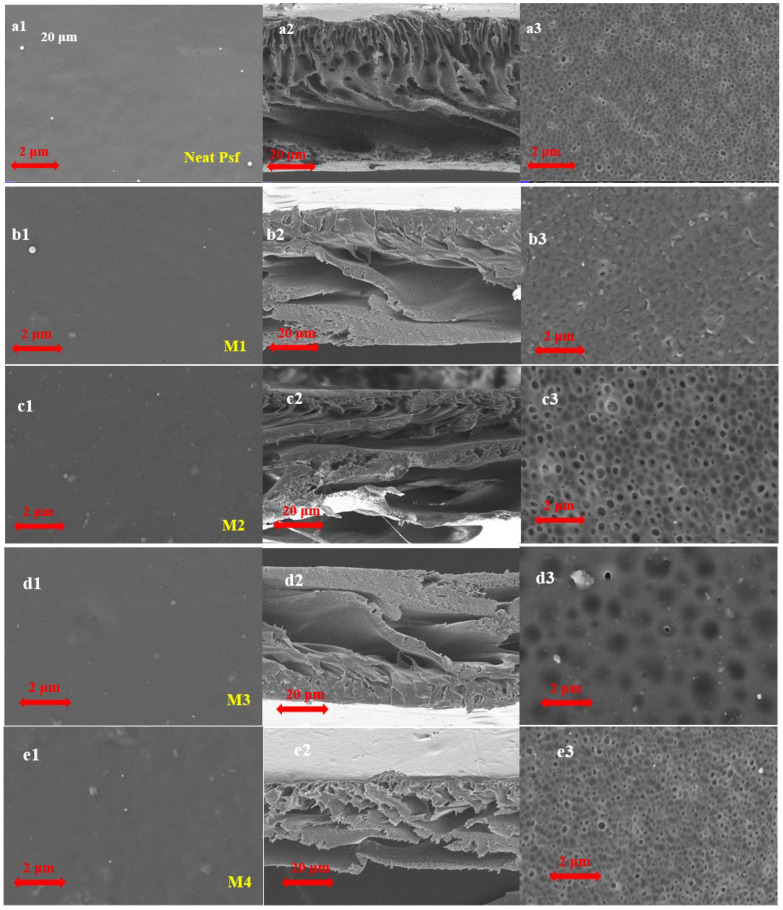

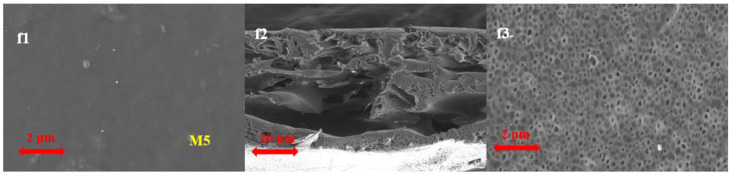
Top surface and cross-section SEM images of PSF–MMT membranes prepared with different compositions of MMT, (**a**) (Neat PSF); (**b**) M_1_ (with 0.5 wt% of MMT); (**c**) M_2_ (with 1 wt% of MMT); (**d**) M_3_ (with 1.5 wt% of MMT); (**e**) M_4_ (with 2 wt% of MMT), and (**f**) M_5_ (with 2.5 wt% of MMT). 1: Top surface; 2: Cross-section; 3: Bottom section.

The surface and cross-section morphologies of the prepared membranes were examined using SEM (see Figure 8). All membranes displayed a characteristic asymmetrical structure, with a dense top layer and a porous sublayer (see Figure 8). The pristine membrane had a smooth, compact surface with limited porosity and an asymmetric cross-section featuring finger-like macrovoids, as shown in Figure 7. Membranes M_1_ and M_2_ developed surface pores along with larger, more prominent finger-like structures. Among all samples, M_3_ showed the highest surface porosity, with well-connected macrovoids, indicating rapid phase inversion during fabrication. Its surface morphology exhibited uniformly distributed circular pores, suggesting improved permeability potential. In contrast, M_4_ and M_5_ demonstrated moderate porosity and relatively denser structures. Notably, M_5_ features a thin skin layer covering the finger-like substructure. Overall, these morphological improvements, especially in M_2_ and M_3_, are likely to enhance membrane performance, particularly for high-flux and selective separation applications.

**Figure 9:**
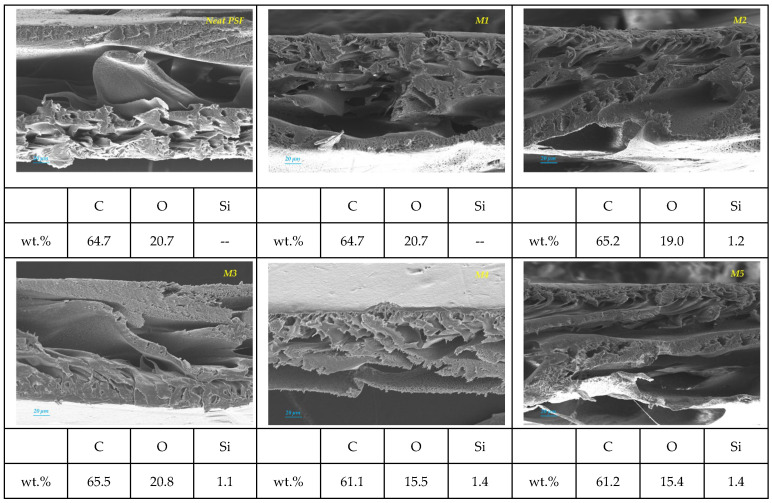
Elemental microanalyses and EDS pattern of the different samples: Neat PSF, M_1_ (MMT-0.5%wt), M_2_ (MMT-1%wt), M_3_ (MMT-1.5%wt), M_4_ (MMT-2%wt), and M_5_ (MMT-2.5%wt). Elements analyzed: carbon (C), silicon (Si), and oxygen (O).

Elemental microanalysis using energy-dispersive X-ray spectroscopy (EDS) was carried out to analyze the elemental composition of the polysulfone (PSF) and mixed matrix membranes. The EDS spectra and corresponding SEM images (Figure 9) reveal the presence and distribution of key elements: carbon (C), oxygen (O), and silicon (Si). The neat PSF membrane showed a high carbon content (64.7 wt%) and a significant oxygen level (20.7 wt%), typical of its aromatic and sulfone backbone. As expected, no silicon was detected in the neat membrane, confirming its unmodified state.

The MMMs displayed silicon content (ranging from 1.1 to 1.4 wt%). These results demonstrate successful incorporation of silicon-based additives into the membrane matrix. The presence of silicon in these samples provides direct evidence of surface modification and suggests potential improvements in membrane hydrophilicity, fouling resistance, and mechanical stability (see Figure 9).

EDS analysis confirmed successful chemical modification of the PSF membranes with MMT, which corresponded to SEM-observed morphological changes. Modified membranes exhibited more pronounced finger-like pores and increased macrovoid formation, likely enhancing water permeability and overall functional performance in future evaluations.

Table 2 presents the mechanical properties of the prepared membranes. The addition of MMT improved the mechanical flexibility of the membranes, with tensile strain at break rising from 21.73% for the neat PSF membrane to a maximum of 38.4% for M_4_. This enhancement is due to the reinforcing effect and good dispersion of MMT clay within the polymer matrix [52]. Although tensile strength remained relatively stable across different formulations, the highest value (4.47 MPa) was observed at low MMT content (M_1_), indicating effective stress transfer at lower filler levels [53]. Young’s modulus varied with composition, with M_2_ exhibiting the highest stiffness (106 MPa), indicating enhanced rigidity at moderate MMT loading. Additionally, an increase in the clay content to 2.5 wt% led to a decline in mechanical properties, likely due to particle aggregation and poor dispersion. These results suggest that optimal MMT incorporation can simultaneously improve the ductility and mechanical strength of the membranes.

Porosity and SEM analyses highlight the significant influence of montmorillonite (MMT) on the structure and performance of PSF membranes. The addition of MMT increased both the average pore radius and overall porosity (Table 3), which is attributed to the hydrophilic character of the clay promoting a more rapid solvent–non-solvent exchange during the phase inversion process.

SEM images confirmed that membranes M_3_ exhibited the largest and most uniform pores, corresponding to enhanced water transport and reduced fouling [52]. However, when the MMT content exceeded 1.5 wt%, particle agglomeration occurred. These aggregates reduced the effective surface area and partially blocked the membrane pores, as observed in M_4_ and M_5_ membranes. Consequently, the pore radius and overall porosity decreased, and the membranes exhibited a higher contact angle, indicating reduced hydrophilicity [22]. Despite these limitations at high loadings, SEM micrographs still revealed a relatively well-dispersed porous network, demonstrating that careful control of MMT concentration is critical to optimizing both morphology and functional performance. Overall, these findings show that MMT incorporation can significantly improve membrane structure and water transport properties, provided that aggregation is avoided [54].

#### 3.2.4. Chemical Resistance

**Figure 10:**
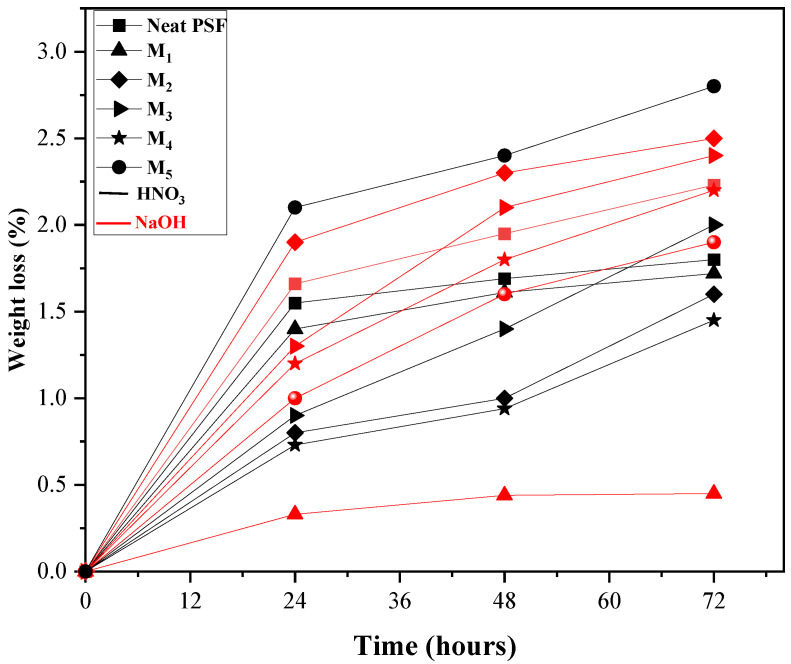
Weight loss percentage versus time of the prepared membranes after immersion in HNO_3_ and NaOH for 3 days at room temperature.

Chemical cleaning of membranes is an accepted method to reduce flux decline and fouling. Among various cleaning techniques, chemical cleaning is the most common. Acidic agents are used to remove inorganic contaminants, while alkaline agents target organic contaminants, including microorganisms. However, chemical cleaning can significantly impact the physical and chemical properties of the membrane, causing surface damage and loss of selectivity. Therefore, chemical resistance in acidic and alkaline conditions was assessed. The percentage of weight loss over time is shown in Figure 10. After three days (72 h), the weight loss ranged from 0.25% to 2.5% across the different membranes. Among the tested samples, the M_2_ membrane exhibited the highest stability in alkaline solution, whereas the M_4_ membrane showed superior resistance in acidic medium.

#### 3.2.5. Hydrophilicity and Water Permeability

**Figure 11:**
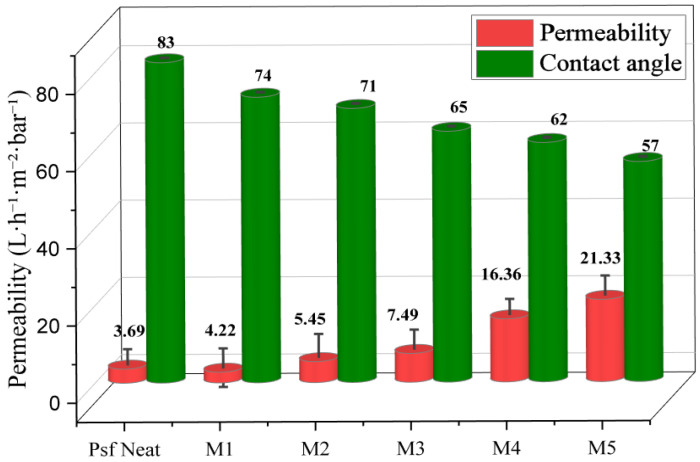
Pure water permeability and static contact angle of the MMT–PSF membranes.

Hydrophilic membranes are commonly used in wastewater treatment because they have higher water flux and are less prone to fouling [55]. Adding montmorillonite (MMT) into the PSF matrix greatly increased the surface hydrophilicity of the membranes, as shown by static water contact angle measurements (Figure 11). The pure PSF membrane showed a contact angle of 83°, reflecting its hydrophobic character. Incorporation of MMT progressively reduced the contact angle, reaching 57° for membrane M_5_, indicating enhanced hydrophilicity.

This effect is attributed to the migration of MMT clay to the membrane surface during phase inversion. This migration, driven by exposure to water (the non-solvent), is facilitated by the hydrophilic nature and negative surface charge of MMT [56]. This process, commonly referred to as “fining,” leads to the accumulation of MMT at the water–membrane interface, enhancing surface wettability. Consequently, the increased hydrophilicity results in a significant improvement in pure water permeability.

The water permeability of the unmodified PSF membrane was 4 L·m^−2^·h^−1^·bar^−1^, whereas the M_5_ membrane exhibited the highest permeability at 21.33 L·m^−2^·h^−1^·bar^−1^. This trend was consistent across all modified membranes (M_1_–M_5_), underscoring the beneficial effect of MMT on both surface properties and water transport efficiency.

### 3.3. Application on DCF Removal, BSA Rejection, and Antifouling Properties

**Figure 12:**
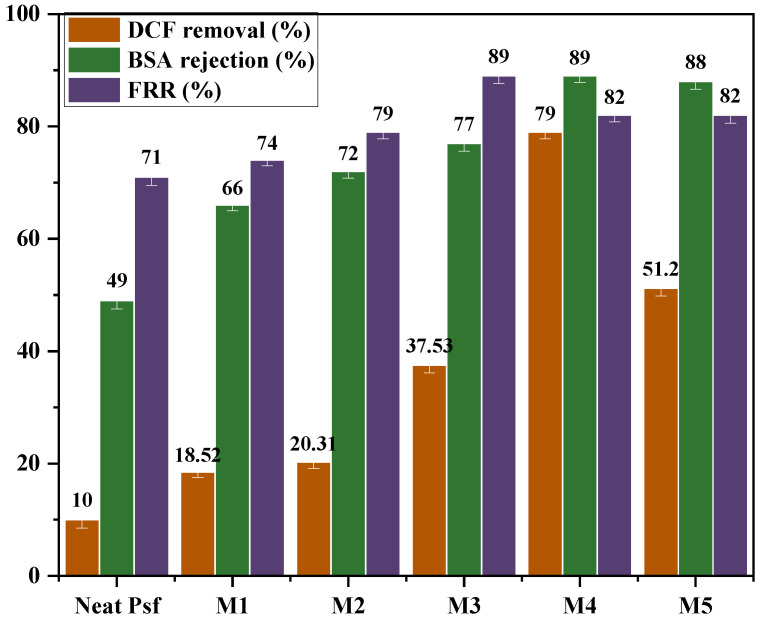
Diclofenac, BSA rejection, and antifouling FRR of the PSF membrane with various MMT contents. (Operating pressure = 10 bar, pH = 7 ± 0.2, initial DCF concentration = 20 mg·L^−1^, feed temperature = 25 °C).

The influence of montmorillonite (MMT) incorporation into polysulfone (PSF) membranes on diclofenac (DCF) and bovine serum albumin (BSA) removal efficiencies, as well as on the flux recovery ratio (FRR), is illustrated in Figure 12.

All experiments were conducted under controlled operating conditions: a transmembrane pressure of 20 bar, pH 7 ± 0.2, and an initial diclofenac (DCF) concentration of 20 mg·L^−1^, and a feed temperature of 25 ± 0.5 °C. The pristine PSF membrane showed modest performance, achieving 10% DCF removal, 49% BSA rejection, and 71% FRR.

The incorporation of MMT led to a significant improvement in the membrane performance. Specifically, DCF removal gradually increased with MMT loading, reaching a maximum of 79% for membrane M_4_. This improvement is due to the higher hydrophilicity and additional adsorption sites provided by MMT, which enhance the interactions between DCF molecules and the membrane matrix. However, at higher loading (membrane M_5_), DCF removal dropped to 51.2%, probably because MMT agglomeration disrupted membrane structure and lowered separation efficiency.

Similarly, BSA rejection increased from 49% (neat PSF) to 89% for membranes M_3_ and M_4_, reflecting a tighter membrane structure and improved size exclusion capabilities. This improvement is likely due to well-dispersed MMT particles modifying the pore structure and reducing macrovoid formation. A slight decrease to 88% for M_5_ suggests that excessive filler content can compromise structural uniformity [22].

The antifouling performance, as indicated by the FRR, also showed significant improvement. FRR increases from 71% for the neat PSF membrane to 89% for M_3_ and remains high at 82% for both M_4_ and M_5_. The enhanced antifouling behavior is due to the increase in hydrophilicity and a smoother surface morphology provided by the MMT, which decreases foulant adhesion and improves membrane cleanability [22].

Overall, membrane M_4_ demonstrated the most balanced performance by achieving high DCF removal (79%), excellent BSA rejection (89%), and strong antifouling properties (FRR = 82%). These results indicate that there is an optimal level of MMT loading, as excessive amounts could lead to negative morphological changes that diminish the benefits of MMT addition.

### 3.4. Diclofenac (DCF)Removal Mechanism by PSF/MMT Composite Membranes

The removal mechanism of diclofenac (DCF) using mixed matrix membranes (MMMs) based on polysulfone incorporating montmorillonite (MMT) involves a synergistic combination of physical and physicochemical processes. Initially, DCF in aqueous solution—mainly in its ionized form (carboxylate anions at neutral pH)—is partially retained through physical sieving as it passes through the membrane pores. This filtration is governed by the molecular size of DCF (296 Da) and the microporous structure of the membrane, which is enhanced by the presence of MMT.

In addition to this mechanical retention, a significant portion of DCF is removed through specific adsorption onto the layered structure of MMT. Montmorillonite provides a high specific surface area and numerous surface functional groups (–OH, Al–OH, Si–O–Si), which enable strong interactions with DCF molecules through hydrogen bonding, electrostatic attraction, and π–π interactions [57]. These interactions promote the immobilization of DCF at the clay–polymer interface, thereby enhancing the membrane’s overall removal performance.

Several studies have confirmed that DCF removal by MMT mainly occurs through adsorption mechanisms, facilitated by the interlayer spacing and surface hydroxyl groups of the clay [58,59]. These findings align with this study’s results, where adding MMT improved both DCF rejection and antifouling performance without significantly impacting membrane permeability. Additionally, recent reviews highlight adsorption as a highly effective and cost-efficient strategy for removing DCF from water [60].

Recent studies on polysulfone/MMT mixed matrix membranes have demonstrated that these synergistic interactions lead to effective DCF adsorption while maintaining high water permeability and antifouling performance [59]. Moreover, adsorption studies of diclofenac on montmorillonite highlight the importance of interlayer spaces and surface hydroxyl groups in capturing DCF molecules, confirming the key role of adsorption over size exclusion mechanisms [60].This dual mechanism of filtration and adsorption is further promoted by the increased hydrophilicity of the membrane resulting from MMT incorporation, which enhances its affinity toward polar organic compounds such as DCF. Consequently, PSF/MMT membranes achieve a high DCF removal efficiency (up to 79%) while maintaining excellent water permeability and antifouling resistance.

**Figure 13:**
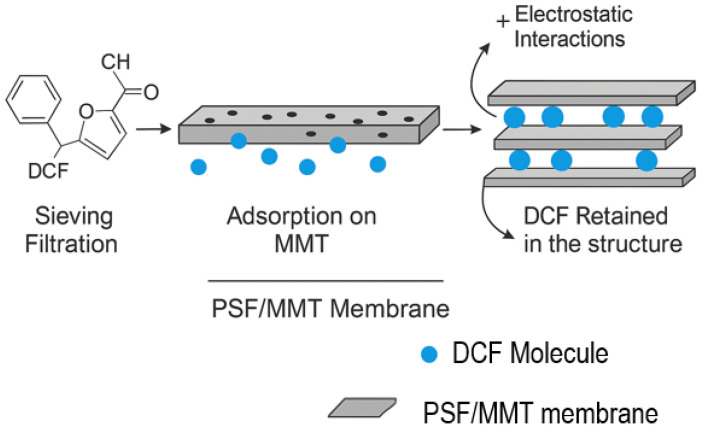
Schematic representation of the diclofenac (DCF) removal mechanism by PSF/MMT composite membranes.

As shown in Figure 13, electrostatic attraction between DCF anions and the polar hydroxyl sites of MMT, combined with hydrophobic π–π stacking, facilitates strong immobilization of DCF molecules within the membrane matrix.

## 4. Comparative Analysis of Literature Data for DCF Removal Using PSF-Based Membranes

**Figure 14:**
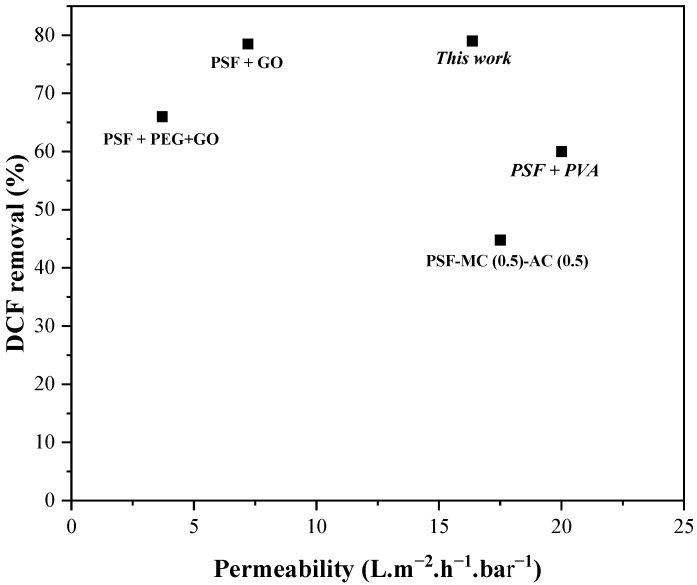
Comparative Analysis of Literature Data for DCF Removal using PSF-Based Membranes. PSF: polysulfone; GO: graphene oxide; PVA: polyvinyl alcohol; PSSMA: polyelectrolyte poly (4-styrenesulfonic acid-co-maleic acid), MC: methylcellulose; AC: activated carbon; PEG: polyethylene glycol; MMT: montmorillonite clay.

Figure 14 compares various polymeric and mixed matrix membranes (MMMs) prepared by the phase inversion method for diclofenac (DCF) removal. In a recent study, we developed a PSF–GO nanocomposite membrane (PSF: 13 wt%, GO: 0.5 wt%, NMP: 86.5 wt%), which exhibited a permeate flux of 7.2 L·m^−2^·h^−1^·bar^−1^ and achieved a high DCF removal efficiency of 78.5%, outperforming most reported PSF-based membranes. This enhanced performance was attributed to the incorporation of GO, which significantly improved the hydrophilicity, surface activity, and antifouling behavior of the membrane [21].

In comparison, a PSF–PEG–GO membrane (PSF: 13 wt%, PEG: 2 wt%, GO: 0.5 wt%) reported in previous work exhibited a moderate DCF removal efficiency of 66%, which was lower than that of the PSF–GO membrane but still demonstrated the beneficial effect of GO incorporation on adsorption capacity. In this system, PEG acted as a pore-forming agent, enhancing both porosity and flux [21].

The PSF–MC/AC (0.5) membrane reported by Nadour et al. [17] exhibited a higher flux (17.5 L·m^−2^·h^−1^·bar^−1^) but a lower removal efficiency (44.7%), due to the limited interaction of activated carbon with DCF in aqueous media. Similarly, the PSF–PVA/PSSMA membrane (PSF: 16 wt%, PVA: 1 wt%, PSSMA: 1 wt%) showed a moderate removal efficiency (60%) and a loose nanofiltration behavior, but it was prone to fouling despite its ability to be chemically regenerated using NaOH [61].

In the present study, the PSF–MMT membrane achieved a water permeability of 16.36 L·m^−2^·h^−1^·bar^−1^ and a DCF removal efficiency of 79%, attributed to the improved membrane morphology and surface properties induced by montmorillonite incorporation, which enhanced both hydrophilicity and adsorption capacity.

The developed PSF–MMT membranes demonstrate an excellent balance between high contaminant removal efficiency, adequate permeation flux, and strong antifouling resistance, highlighting their potential for practical application in pharmaceutical wastewater treatment.

## 5. Conclusions

This study demonstrated that incorporating montmorillonite (MMT) into polysulfone (PSF) membranes significantly enhances their performance in water treatment, particularly for the removal of pharmaceutical contaminants such as diclofenac (DCF). At the optimal MMT loading of 2 wt%, the composite membranes exhibited improved water permeability (16.36 L·m^−2^·h^−1^·bar^−1^), effective DCF removal (79%), and excellent antifouling properties. When tested with bovine serum albumin (BSA) as a model foulant, the M_4_ membrane achieved a high rejection rate (89%) and a flux recovery ratio (FRR) exceeding 82%. However, at higher MMT concentrations (2.5 wt%), particle aggregation occurred, adversely affecting permeability, rejection, and fouling resistance. These findings highlight the importance of optimizing the filler content to achieve a balanced combination of flux, selectivity, and structural stability.

Overall, PSF/MMT nanocomposite membranes show strong potential for advanced water purification applications, offering a durable and efficient approach for mitigating pharmaceutical pollutants in wastewater. Future work will focus on scaling up the PSF/MMT system for continuous-flow applications and testing with real pharmaceutical wastewater.

## Figures and Tables

**Table 1:** Membrane PSF–MMT composition.

Samples	PSF (wt%)	MMT (wt%)	NMP (wt%)
Neat PSF	13	0.0	87.0
M_1_(MMT-0.5%wt)	13	0.5	86.5
M_2_(MMT-1%wt)	13	1.0	86.0
M_3_(MMT-1.5%wt)	13	1.5	85.5
M_4_(MMT-2%wt)	13	2.0	85.0
M_5_(MMT-2.5%wt)	13	2.5	84.5

**Table 2:** Effect of MMT content on tensile strength (MPa), tensile strain at break (%), and Young’s modulus of the different prepared membranes.

Membrane’s Types	Tensile Strain at Break (%)	Tensile Strength (MPa)	Young’s Module (MPa)
Neat PSF	21.73 ± 1.18	3.97 ± 1.00	93.65 ± 1.04
M_1_ (MMT-0.5%wt)	29.43 ± 1.02	4.47 ± 1.05	70.08 ± 1.20
M_2_ (MMT-1%wt)	30.91 ± 1.17	3.61 ± 1.08	106 ± 1.02
M_3_ (MMT-1.5%wt)	37.45 ±1.35	3.44 ± 1.11	97.64 ± 1.02
M_4_ (MMT-2%wt)	38.40 ±1.20	3.80 ± 1.05	94.52 ± 1.16
M_5_ (MMT-2.5%wt)	35.95 ±1.08	3.50 ± 1.05	91.62 ± 1.05

**Table 3:** Mean pore size and porosity of the PSF–MMT membranes.

Membrane’s Types	Mean Pore Radius r (nm)	Porosity (%)
Neat PSF	16.7 ± 0.6	74 ± 0.6
M_1_ (MMT-0.5%wt)	18.9 ± 0.4	80 ± 0.8
M_2_ (MMT-1%wt)	22.4 ± 0.8	86 ± 0.7
M_3_ (MMT-1.5%wt)	24.4 ±0.5	89 ± 0.9
M_4_ (MMT-2%wt)	19.0 ± 0.7	82 ± 0.6
M_5_ (MMT-2.5%wt)	21.0 ±0.6	85 ± 0.8

## Data Availability

The data presented in this study are available on request from the corresponding authors.
